# Beyond Precision: Ambiomic Survivorship in Childhood and AYA Cancer

**DOI:** 10.3390/cancers18010007

**Published:** 2025-12-19

**Authors:** Juan Antonio Ortega-García, Omar Shakeel, Nicole M. Wood, Antonio Pérez-Martínez, Jose Luís Fuster-Soler, Mark D. Miller

**Affiliations:** 1 Pediatric Environmental Health Specialty Unit, Department of Pediatrics, Hospital Clínico Universitario Virgen de la Arrixaca (HCUVA), 30120 Murcia, Spain; 2 Pediatric Hemato-Oncology, Clinical Hospital University Virgen Arrixaca, Instituto Murciano de Investigación Biosanitaria (IMIB) Pascual Parrilla, University of Murcia, 30120 Murcia, Spain; josel.fuster@carm.es; 3 Texas Children’s Cancer and Hematology Center, Baylor College of Medicine, Houston, TX 77030, USA; shakeel@bcm.edu; 4 MidAmerica Pediatric Environmental Health Specialty Unit, Children’s Mercy Kansas City, Kansas City, MO 64108, USA; nwood@cmh.edu; 5 Pediatric Hemato-Oncology Department, IdiPAZ-CNIO Pediatric Oncology Clinical Research Unit Hospital University La Paz, 28046 Madrid, Spain; aperezmartinez@salud.madrid.org; 6 Western States Pediatric Environmental Health Specialty Unit, University of California, San Francisco, CA 94143, USA; mark.miller2@ucsf.edu

**Keywords:** pediatric cancer, survivorship care model, AYA oncology, exposome, ambiomic medicine, environmental health, late effects, chronic diseases, equity

## Abstract

**Background:** Survival among children and adolescents and young adults (AYA) with cancer has improved substantially over recent decades; however, dominant survivorship models remain reactive—activated post-treatment and anchored to static exposure- and organ-based screening. This design underuses the anticipatory window at diagnosis and overlooks environmental and social determinants that modulate outcomes across the life course. **Methods:** We narratively reviewed international frameworks including the Children’s Oncology Group (COG), the International Late Effects of Childhood Cancer Guideline Harmonization Group (IGHG), the Pan-European Network for Care of Survivors after Childhood and Adolescent Cancer (PanCare) and the National Comprehensive Cancer Network (NCCN), and synthesized evidence on environmental determinants, exposomics, toxicogenomics, and implementation. Building on two decades of real-world practice, we describe the evolution from the Pediatric Environmental History (PEHis) to the Ambiomic Health Compass (AHC), integrating genomic, exposomic, geospatial, clinical, and biomonitoring layers into routine care. In this framework, survivorship is conceptualized as beginning at the time of cancer diagnosis (“day 0”). **Results:** PEHis operationalizes guideline-based care with structured environmental and social assessment, personalized plans, and community integration, contributing to improved survival, healthier behaviors, reduced treatment-related mortality and stronger oncology–primary-care coordination. AHC extends PEHis with dynamic risk recalibration, contextual alerts, targeted biomonitoring, and toxicogenomic interpretation, enabling anticipatory decisions from day 0. The manuscript summarizes the paradigm shift (current vs. Ambiomic models), the domain-specific expansion over existing guidelines, the core clinical/system tools, and time-bound metrics (12, 24, 60 months) to support implementation and evaluation. **Conclusions:** Survivorship should move upstream—from late surveillance to ambiomic, exposure-aware care beginning at diagnosis. Integrating advanced exposomics, mutational epidemiology, and explainable analytics can reduce preventable events and chronicity, enhance equity, and align pediatric oncology with planetary health. The PEHis–AHC continuum offers a scalable blueprint for next-generation survivorship programs in Europe and beyond. Ambiomic medicine does not replace precision medicine—it completes and extends it by integrating exposomics, social context, and anticipatory analytics from day 0.

## Simple Summary

Survival after childhood and adolescent and young adult (AYA) cancer has improved markedly, but many survivors still face serious complications, chronic health problems and social inequalities. Most survivorship programs begin after treatment and focus on organs and chemotherapy doses, rather than on the real environments in which children live. In this review, we describe an “ambiomic” survivorship model that starts at the day of diagnosis (day 0) and follows children and families across their whole lives. The model combines medical information with structured genomic, exposomic, environmental and social data, and uses and expands international survivorship guidelines by adding layers on air pollution, housing, school, lifestyle and social conditions, through tools such as the Pediatric Environmental History and the Ambiomic Health Compass. These tools help anticipate preventable complications, reduce treatment-related deaths, support healthier behaviors and improve quality of life. We also show how new science on environmental exposures and cancer biology can help make prevention and prognosis more precise. All of this is framed along the cancer continuum within a global and planetary health perspective, recognizing that children with cancer are affected not only by their genes and treatments, but also by the quality of the environments and societies in which they grow up. Our proposal offers a practical roadmap for health systems to build fairer, more proactive survivorship care for children and AYA with cancer in Europe and beyond.

## 1. Introduction

Increased survival after childhood and adolescent and young adult (AYA) cancer represents one of the most remarkable achievements in modern medicine. In high-income countries, more than 80% of children now survive five years after diagnosis—a milestone that has transformed cancer from an acute, often fatal disease into a chronic condition with lifelong implications [1]. Yet this success is uneven. In many low- and middle-income countries, survival remains below 40%, and even in advanced health systems [2,3], treatment-related mortality (TRM)—often driven by preventable infections during therapy—accounts for up to 20% of deaths [4]. Moreover, survivors continue to face excess mortality and chronic health burdens decades after diagnosis, highlighting both the progress made and the limits of current approaches [1,2,3,4]. This aligns with recent European and global strategic analyses calling for a systemic transformation of childhood cancer care and survivorship by 2030, emphasizing equity, prevention, and environmental determinants [5].

Survivorship today is too often treated as a post-treatment chapter—a period of late-effect surveillance that begins after therapy ends. This reactive model, organ-based and dose-specific screening, has brought structure and safety but no longer matches the complexity of the survivor’s journey. Building on this, the current risk architecture remains anchored to static, exposure- and organ-based screening, limiting its ability to anticipate dynamic, real-world determinants of survivorship [6,7,8,9]. A model that begins late and fragments during the transition to adult care cannot fully prevent avoidable harm, reduce relapse risk, or address persistent inequities. Recent analyses from across Asia reinforce this global pattern: survivorship programs remain highly uneven, largely oncologist-centered, and rarely integrate mental health, education, or social and environmental determinants. These findings highlight that fragmented, post-treatment survivorship is not only a Western limitation but a worldwide structural gap, underscoring the need for earlier, anticipatory, and context-sensitive models [10]. A comprehensive meta-analysis of nearly 400,000 childhood cancer survivors reported significantly lower educational attainment, markedly higher health-related unemployment and reduced rates of marriage and parenthood compared with the general population—underscoring that survivorship care must extend into lifelong social and economic reintegration [11].

It is time to move survivorship upstream [12,13,14]. Childhood and AYA cancer should be approached as a chronic, vulnerability-sensitive process that begins at diagnosis, integrating prevention, early intervention, and long-term adaptation [15,16]. Ambiomics—an integrative framework linking genomic, exposomic, behavioral, social, and socio-ecolo-gical determinants—offers a way to transform follow-up into real-time navigation of risk and opportunity, rather than a static checklist of late effects [17,18,19,20]. This integrative logic aligns with and expands the syndemic framework, originally articulated by Singer, which emphasizes how biological vulnerability interacts synergistically with adverse social and environmental conditions. In childhood and AYA cancer, treatment-related toxicities, immunosuppression, socioeconomic disadvantage, and environmental exposures frequently cluster and reinforce one another, producing outcomes more severe than any single factor in isolation. Ambiomic survivorship operationalizes this syndemic perspective by integrating structured clinical tools, exposomic intelligence, and dynamic risk recalibration from diagnosis, allowing these interacting vulnerabilities to be identified, monitored, and mitigated throughout the survivorship continuum [21,22].

Building on the Pediatric Environmental History (PEHis), already operational in clinical practice to capture modifiable environmental and social factors [5,6,7,23], we propose the Ambiomic Health Compass (AHC) as a new layer of survivorship care. This framework integrates biomarkers, geospatial context, and explainable analytics to guide anticipatory interventions from day 0 (diagnosis) [18,19,24,25]. The goal is not to replace existing guidelines, but to reframe survivorship as an active, patient–family–community process that complements and amplifies them. This framework explicitly extends precision medicine beyond the genome by adding real-world exposomic and social layers that drive anticipatory, context-aware decisions.

This article aims to present a transformative, ambiomic framework for childhood and AYA cancer survivorship. Starting at diagnosis (day 0), it seeks to shift follow-up from static surveillance to dynamic, preventive care—reducing TRM, preventing relapse and morbidity, improving quality of life, and providing an equitable, scalable roadmap for implementation in Europe and beyond.

## 2. Methods and Approach

This review was conducted using a structured narrative synthesis approach designed to integrate conceptual, clinical, environmental, and implementation evidence.

First, we performed a comparative analysis of major international survivorship frameworks, including the Children’s Oncology Group (COG) Long-Term Follow-Up Guidelines, the International Late Effects of Childhood Cancer Guideline Harmonization Group (IGHG) recommendations, the Pan-European Network for Care of Survivors after Childhood and Adolescent Cancer (PanCare) framework, and the National Comprehensive Cancer Network (NCCN) guidance for AYA Oncology. This analysis was used to identify convergent principles, structural gaps, and opportunities for earlier, context-aware integration.

Second, we conducted targeted literature searches in PubMed/MEDLINE, Scopus, Web of Science, and Google Scholar using predefined keywords related to survivorship, exposomics, toxicogenomics, environmental determinants, social determinants of health, health services research, and implementation science. The most recent search was completed in September 2025.

Third, we reviewed specialized sources—including the Comparative Toxicogenomics Database (CTD), environmental exposure datasets, indoor air quality modeling tools, and reports from the World Health Organization (WHO) and the United Nations Environment Program (UNEP)—to map gene–environment–context interactions relevant to childhood and AYA cancer survivorship.

Fourth, we incorporated two decades of real-world operational experience from the Pediatric Environmental History (PEHis) program and its expansion into the Ambiomic Health Compass (AHC), including pathway redesign, navigation models, dynamic risk stratification, patient-reported outcomes (PROs), patient-reported experience measures (PREMs), and exposure-aware interventions.

Finally, we synthesized all sources using a framework-based approach to describe how genomic, exposomic, developmental, environmental, and social determinants interact across the survivorship continuum. The objective was to generate an integrative conceptual model rather than a systematic review.

## 3. Current Landscape of Survivorship Programs

Over the past two decades, survivorship programs have evolved from fragmented practices into structured, evidence-based frameworks. In the United States, the Children’s Oncology Group Long-Term Follow-Up Guidelines (COG LTFU) remain the reference standard for screening and management of late effects. In its most recent version (6.0, 2023), the guideline includes 165 sections and 45 “health links” covering genetic predisposition, novel therapies, vaccination, and organ-specific late effects [26]. These guidelines organize follow-up around therapeutic exposures (e.g., chemotherapy, radiotherapy) and target organs, defining screening schedules and patient education strategies. Digital platforms such as Passport for Care and SurvivorLink have translated these guidelines into individualized, risk-based follow-up plans accessible to patients and clinicians alike [27,28,29].

The AYA field has also gained visibility through the NCCN Guidelines for AYA Oncology, which highlight fertility, mental health, lifestyle risk behaviors, access to clinical trials, and structured transitional care [30]. In Europe, PanCare and its initiatives (PanCareSurFup and PanCareFollowUp) have contributed organizational and clinical guidance [31,32], while the IGHG has aligned disease-specific recommendations globally, including standardization of cardiomyopathy surveillance thresholds across anthracycline- and radiotherapy-exposed survivors [33]. Collectively, these frameworks have standardized language, increased patient health and safety, improved early detection of late effects, and strengthened the transition to adult care in many countries.

Cancer predisposition clinics serve as an upstream entry point. In addition to guideline-based survivorship care [34], many centers now run cancer predisposition clinics that identify and follow children and AYAs with germline cancer syndromes (e.g., TP53/Li-Fraumeni, RB1, DICER1, NF1, mismatch-repair defects, etc.) [35,36]. For these patients, survivorship effectively begins before diagnosis. Care plans blend genetic and inheritance counseling, cascade testing, and MRI-based, radiation-sparing surveillance where indicated [37], together with psychosocial counseling, and structured transition to adult provider with expertise in predisposition surveillance (or to primary care when appropriate) [38].

These clinics provide an ideal on-ramp to ambiomic survivorship, where environmental and social layers are integrated early, alongside genomic information, to reduce avoidable exposures, optimize screening choices, and support family-level prevention. Timely identification by genetic counselors is essential to route patients to cancer predisposition clinics, enabling early initiation of exposure-aware surveillance and family-based cascade testing. For example, air pollution, particularly fine particulate matter (PM2.5) and traffic-related pollutants, has been associated with increased risk of certain pediatric cancers and adverse cancer outcomes [9,39,40]. Integrating local air quality data and patient exposure histories into routine care can help personalize surveillance strategies and inform anticipatory guidance in predisposed populations. Alongside international frameworks, regional innovation has also shaped the field. A landmark example is the Environmental and Community Health Program for long-term follow-up survivor pediatric cancer (PLASESCAP-MUR, Murcia, Spain), developed by Pediatric Environmental Health Specialty Unit (PEHSU) since 2003 and recognized as a Good Practice by the Spanish Ministry of Health (Cancer Strategy of the Spanish National Health System, 2006; p. 180) [41]. PLASESCAP pioneered the systematic integration of PEHis into oncology follow-up, bridging hospital care, primary care, public health, and the community [5,23]. Its practical tools—the Green Page and Green Passport—enable clinicians to identify modifiable exposures and anticipate late effects, supporting personalized survivorship plans. Documented outcomes include lower indoor toxicant exposure, reduced smoking prevalence among survivors, improved metabolic profiles, and stronger care coordination [6,7,23]. PLASESCAP is widely regarded as the seed model for the ambiomic survivorship framework (PEHis/AHC) now being scaled through collaborations with major European centers such as Hospital La Paz (Madrid), Sant Joan de Déu (Barcelona) and Charité (Berlin), aligning with the Horizon Europe Mission Cancer agenda.

Parallel efforts are emerging in the United States, where colleagues from the Childhood Cancer and the Environment Program (CCEP) of the PEHSUs are developing environmental consultative services for children with cancer predisposition syndromes and childhood cancer survivors within clinical pediatric oncology programs [42]. This initiative aims to systematically address environmental risk factors—air pollution, tobacco smoke, pesticides, and climate-related stressors—across the oncology care continuum, offering evidence-based counseling and mitigation strategies that can reduce morbidity and mortality [PEHSU—Childhood Cancer & the Environment: National Program] [43]. Surveys of attending physicians, advanced practice providers, social workers, and research personnel at two large pediatric oncology centers found strong support for developing and using such environmental health consultative service [20,42].

Despite these advances, most programs rely on exposure-based, largely static risk tiers post-treatment, missing the anticipatory window and leaving evolving vulnerabilities under-addressed [44]. The anticipatory ambiomic window preceding and at diagnosis constitutes a pivotal moment in which children and AYA exhibit heightened vulnerability to acute treatment toxicity and to modifiable ambiomic factors—environmental, social, and exposomic—that shape the initial Day-0 risk architecture [5,6,7,8,9,20,44,45]. The current structure also fails to address environmental and social factors that by themselves are also associated with the same spectrum of unfavorable outcomes as oncologic treatment regimens [46]. Furthermore, fragmentation between pediatric and adult care continues to generate losses to follow-up, and socioeconomic, geographic, and migration-related inequities remain unaddressed in many systems and is no longer sufficient to overcome persistent TRM, relapses, and chronic disease burden [47,48,49].

The next leap will require redesigning survivorship as a front-loaded process—beginning prior to or at diagnosis and continuously adapting to evolving biological, environmental, and social vulnerability profiles. This transformation aligns with the environmental–ecosystemic framework proposed by Ortega-García, who conceptualized health, environment, and disease as an inseparable triad and outlined the transition toward anticipatory, planetary health systems [19,24]. This is precisely where the PEHis and the AHC can amplify the impact of existing guidelines, aligning clinical care, primary care, public health, and community actors around the patient.

## 4. Key Limitations and the Path Toward a Transformative Paradigm

Contemporary survivorship guidelines for childhood and AYA cancer have standardized organ- and exposure-based screening and elevated psychosocial needs. As previously discussed, however, the prevailing model remains late and reactive: follow-up is largely post-treatment, risk tiers are exposure-anchored and mostly static, and care fragments across subspecialties with losses to follow up during the pediatric-to-adult transition. Valuable as they are, current frameworks do not capture the dynamic nature of risk or its environmental modulation and the anticipatory window that opens at diagnosis. These constraints help explain the survival plateau seen in recent population-based analyses [1], including nationwide data suggesting that higher ambient PM_2.5_ exposure is associated with poorer survival in childhood cancer [9]. Taken together, these limitations call for a paradigm shift—from late, organ- and dose-based surveillance to an ambiomic, context-aware survivorship model anchored at diagnosis.

Building on these observed limitations, several structural gaps become evident and hinder the ability of current models to anticipate risk and deliver integrated survivorship care. Five structural gaps account for this performance ceiling:

*Timing*. Most interventions start after therapy, forfeiting the broader anticipatory window that should span before, at, and after diagnosis. In practice, for childhood and AYA cancer, this means a critical period from diagnosis through early treatment, when children and adolescents are particularly vulnerable to acute toxicities and to modifiable environmental and social factors, although other windows (e.g., prenatal or post-treatment) also remain relevant [6]. This late timing contributes to TRM during immunosuppression and chemotherapy cycles—an avoidable share of deaths that could be reduced with proactive measures during active treatment. Although existing guidelines mention education and lifestyle, they do not systematically integrate the exposome nor operationalize real-time alerts and responses to everyday chemical, physical, biological, and social risks.

*Static risk architecture*. Current stratification focuses on past doses and target organs, with insufficient weight for dynamic risk that blends clinical data with longitudinal social determinants and environmental layers. Recent studies associate air pollution, secondhand smoke, and residential pesticide use with worse outcomes after pediatric cancer [5,6,7,8,9,50,51], plausibly via oxidative stress, immune dysregulation, and impaired DNA repair [46]. PanCare and IGHG have advanced harmonization, but data interoperability, systematic patient-reported outcomes, and geospatial integration remain heterogeneous across countries and centers, hindering adherence, continuity, and comparability.

*Fragmentation and transition*. NCCN AYA guidelines have highlighted the need for tailored routes and teams, but real-world practice still suffers losses in the pediatric-to-adult handover, uneven coverage, and variable AYA competencies [30]. Equity gaps persist by socioeconomic status, rurality, and migration, with lower surveillance adherence and higher chronic burden in vulnerable groups. Principles of person-centered care are acknowledged, but structural supports (navigation, social prescribing, school/municipality coordination) are inconsistently implemented.

*Operational focus*. Even where European Society for Medical Oncology (ESMO) and others expand survivorship to physical, psychological, social and functional domains [52], operational frameworks still prioritize lists of toxicities and structured screening schedules rooted in exposure-based risk stratification [53]—with limited capacity to reduce TRM, curb avoidable readmissions, limit cumulative diagnostic radiation, or accelerate educational and vocational recovery.

*Implication*. The next quality leap will not come from adding more end-of-line screening but from shifting the center of gravity to the beginning of the journey, integrating clinical and contextual data into auditable, real-time decisions across the childhood → AYA → young adult continuum. This shift can be summarized in the transition from a late, reactive paradigm to an early, ambiomic model anchored at diagnosis (Table 1).

## 5. The Transformative Approach: From PEHis to Ambiomic Development

The natural evolution of survivorship programs is to activate care at diagnosis, treat survivorship as a chronic, vulnerability-sensitive process, and replace static labels with living risk that updates with real life [31]. Practically, this means acting before harm emerges: adjusting prophylaxis; minimizing avoidable diagnostic radiation; improving indoor and school air; eliminating tobacco smoke exposure; mitigating radon where relevant; and supporting sleep, activity, and social needs. Each of these and other environmental risk factors that are associated with cancer outcome risks [19] are also associated with positively impacting the health of the general pediatric population [54,55]. This makes including these factors in guidance for this vulnerable population additionally valuable. With this logic, follow-up ceases to be a calendar and becomes personalized navigation. The pediatric-to-adult transition turns into a guided handover with data continuity, explicit responsibilities on both shores, and the same scaffolding benefiting the AYA stage—not as a silo, but as the logical extension of a journey that began in childhood.

This vision is not theoretical. In 2003, a small team at the Hospital Virgen de la Arrixaca (Murcia, Spain) began systematically integrating environmental and social dimensions into cancer follow-up through the PLASESCAP program [56,57]. What started as a local initiative grew into the PEHis—a structured clinical framework that captures modifiable exposures and social determinants from diagnosis [5,57,58]. Over time, PEHis has demonstrated tangible impact: some of the highest survival rates in Europe, healthier lifestyles, reduced cardiovascular risk, improved metabolic profiles, and stronger coordination between oncology and primary care [23]. Beyond the clinic, it has influenced environmental policies [59], improved indoor air quality, reduced smoking prevalence among survivors, and fostered community engagement [6,7].

What makes PEHis distinctive is not that it replaces existing guidelines, but that it expands and enriches them. It integrates international protocols (COG, IGHG, PanCare, NCCN) and adds a living exposomic layer: air, water, household toxicants, neighborhood stressors, family habits, and social networks. This transforms survivorship from a static follow-up schedule into a dynamic, vulnerability-sensitive journey. This evolution—from guideline foundations to PEHis operationalization and then to AHC’s ambiomic expansion—is summarized in Table 2.

**Figure 1:**
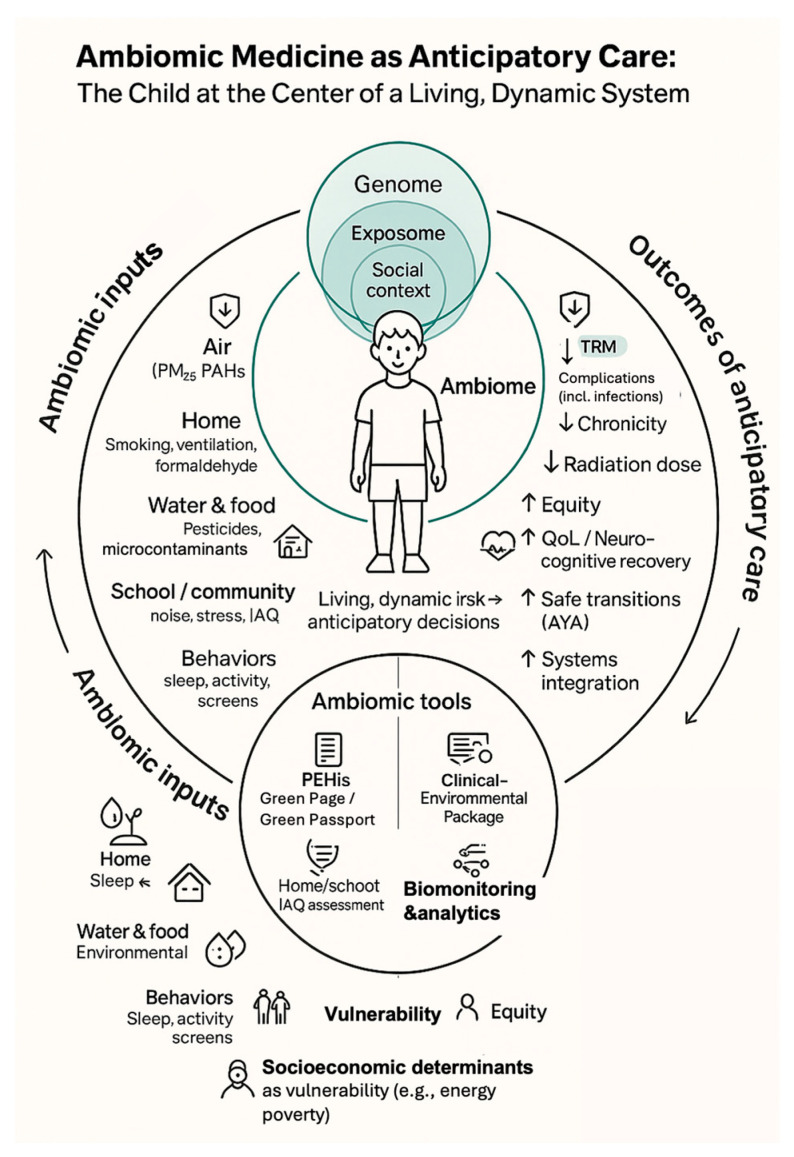
**Ambiomic Anticipatory survivorship model.** Schematic placing the child at the center of an ambiome where genomic, environmental and social determinants converge. Left-hand elements illustrate modifiable domains (air, home, water/food, school/community and behaviors) operationalized through PEHis tools (Green Page/Green Passport), home/school IAQ assessment, clinical–environmental care packages and targeted biomonitoring/analytics. Right-hand elements show anticipated benefits of this anticipatory model, including reduced TRM and complications, lower chronicity and diagnostic radiation, and improved equity, neurocognitive recovery, AYA transitions and systems integration. Arrows represent anticipatory and, where applicable, bidirectional pathways linking ambiomic inputs, vulnerability domains and clinical decision-making, illustrating how modifiable environmental and social determinants are translated into preventive interventions and dynamically recalibrated through feedback from clinical and functional outcomes, beginning at diagnosis (day 0). Abbreviations: TRM, treatment-related mortality; IAQ, indoor air quality; AYA, adolescent and young adult; PEHis, Pediatric Environmental History; AHC, Ambiomic Health Compass; QoL, Quality of life; PAHs, Polycyclic Aromatic Hydrocarbons. Source: Prepared by the authors.

This ambiomic, anticipatory model—linking modifiable environmental and social inputs, PEHis tools, and AHC analytics to downstream clinical and equity outcomes—is schematically depicted in Figure 1, which places the child at the center of a living ambiome.

For families followed in predisposition clinics, PEHis provides actionable context from day zero—home/school IAQ, tobacco smoke, radon, UV and heat exposure, sleep and activity, housing/energy poverty and geographic information systems—while the AHC adds toxicogenomic interpretation (e.g., exposure–gene maps relevant to *TP53* or Li-Fraumeni or *KMT2A*, …), targeted biomonitoring, and radiation-aware surveillance planning. This upstream integration aligns clinical genetics, oncology, and community actors, converting surveillance into anticipatory navigation that is safer (e.g., MRI-first where feasible), more equitable, and family-centered. Building networks with genetic counselors expands early identification, cascade testing, and exposure-aware surveillance pathways—not only radiation but also other carcinogens (e.g., benzene/PAHs, pesticides, tobacco smoke)—providing a natural entry point for ambiomic analytics and family-level prevention.

An essential step in this evolution is the incorporation of molecular environmental intelligence—something PEHis could not originally provide. While PEHis identifies modifiable exposures, geospatial and social determinants, the AHC adds two complementary layers: toxicogenomic interpretation and biomonitoring [25]. Toxicogenomic tools allow clinicians to understand whether specific exposures interact with genes relevant to oncogenesis, treatment response, or toxicity—such as the gene–environment interaction maps described for *KMT2A* [25,60]. Biomonitoring then validates whether these exposures are biologically active in the patient, translating external risks into measurable internal signals. Together, these elements transform environmental information into actionable precision, enabling risk to be recalibrated dynamically and allowing decisions to be personalized beyond the genome [19].

Building on this foundation, the AHC represents the next step [5,20,24,25]. AHC integrates exposomic, genomic, clinical, and geospatial data into a real-time navigational map—a compass for both clinicians and families [61,62,63,64]. Its aim is simple yet powerful: to anticipate preventable complications, tailor interventions, and weave survivorship into the places where children live, learn, and grow, in line with emerging geospatial and environmental cancer frameworks [65,66]. In this sense, ambiomic medicine represents an extension of precision approaches, integrating genome, exposome and context into an anticipatory model of prevention and care [25,62,63]. Ambiomic Medicine expands Precision Medicine by integrating genomic, exposomic, developmental, environmental and psychosocial layers into a unified, anticipatory medical framework.

In clinical practice, this becomes a day-zero plan, an initial risk profile co-created with the family by week two, and a living Green Passport fully operational by day 30, consistent with contemporary survivorship care plan models and risk-based follow-up frameworks [5,31,32,67,68]. Contextual alerts prevent clinical inertia, while navigation teams align hospital, primary care, public health, and schools around a single plan, a shared language, and measurable outcomes, as advocated by shared-care and patient-navigation models in survivorship [69,70,71]. This approach aligns with international standards while advancing toward anticipatory prevention, TRM reduction, chronicity modulation, and equity as explicit success criteria, in line with WHO CureAll and global survivorship frameworks [72,73]. To operationalize this anticipatory model, PEHis–AHC relies on a modular set of clinical and system tools that can be activated from day zero and adapted across contexts (Table 3).

The AHC architecture is designed to integrate classical clinical determinants (cancer type, stage, grading, comorbidities and treatment exposures) with ambiomic inputs to refine anticipatory risk trajectories. This includes the potential incorporation of next-generation multi-omics biomarkers that precede abnormalities in classical tumor markers.

## 6. Evidence of Impact: A Real-World Transformation

The PEHis is not a theoretical construct. Developed and refined over two decades in a complex Mediterranean region with significant environmental and social heterogeneity, it has shown that integrating environmental and social intelligence into survivorship care produces measurable and clinically relevant improvements [5,6,7,23,56,57,58]. Similar approaches are now being piloted in Latin America (EnSuChiCa) [5] and within US PEHSU-based ‘childhood cancer and the environment’ programs [43], which use PEHis tools to guide individualized risk reduction and low-carbon, healthy lifestyle counseling for childhood cancer patients and survivors.

Since its implementation in Murcia, childhood cancer survival has risen from levels comparable to European averages to >85% at five years and close to 80% at ten years—among the highest in Europe [5]. Importantly, these gains have been accompanied by healthier survivor profiles, including lower secondhand smoke exposure, improved metabolic indicators, and stronger adherence to protective behaviors compared with national cohorts [5,6,7].

A notable achievement has been the reduction in TRM. Early identification of modifiable exposures—particularly tobacco smoke, indoor air pollutants, and infection-related risk conditions—has prevented avoidable complications during active therapy, addressing the most persistent and preventable sources of mortality in pediatric oncology [6].

The program has also generated upstream regulatory impact. Aggregated exposure data have contributed to air-quality monitoring improvements, informed tobacco-control policies, and stimulated local environmental action in the regulation of industrial emissions [59,74] and in municipal-level regulation of chemical use [75]. PEHis has additionally fostered new professional roles—environmental health nurses, environmental clinicians, and exposure-data analysts—supporting long-term sustainability within a resource-limited public system. Its adoption across 85 primary care centers demonstrates feasibility and scalability [23]. ENSUCHICA originated as an international extension of the Murcia PEHis–PEHSU survivorship program, created to scale up its environmental and community-based approach to childhood cancer survivorship across Europe and Latin America [5].

Taken together, this combination of improved survival, healthier lifestyles, reduced TRM, community engagement, and system-level integration represents an unprecedented shift in pediatric survivorship care and illustrates how environmental intelligence can function as a structural component of cancer care rather than an adjunct element.

## 7. Opportunities and Challenges: Building the Next Generation of Survivorship Care

From a syndemic perspective, childhood and AYA cancer survivorship is shaped by interacting biological, environmental, and social vulnerabilities rather than by isolated factors. The ambiomic survivorship model operationalizes this perspective by integrating environmental exposures, social determinants, and treatment-related risks into a unified, anticipatory framework. This aligns with contemporary extensions of syndemic theory, which emphasize how interacting vulnerabilities compound inequities [22]. By identifying and interrupting these synergistic pathways from diagnosis, the PEHis–AHC structure addresses the core mechanisms through which syndemics produce disproportionate harm, particularly in socioeconomically or environmentally vulnerable populations.

For any innovation to become transformative, it must navigate the space between promise and practical adoption. The PEHis–AHC model offers a clinically mature and operationally feasible framework, but its capacity to scale lies in how effectively it can be adapted across diverse health systems [68,76].

A key advantage is modularity. PEHis does not require parallel structures; it can be layered onto existing survivorship frameworks. Implementation can begin with a single tool—such as the Green Page—followed by stepwise integration of navigation, environmental measurement, biomarkers, and ambiomic analytics. This enables health systems of varying resource levels and digital maturity to adopt the model progressively [31,67,73].

Another opportunity is its deep alignment with primary care and public health. Rather than concentrating survivorship exclusively in tertiary oncology centers, the model distributes responsibilities across community networks—schools, municipal services, primary care teams—transforming survivorship into a shared societal process. This not only enhances equity but also secures long-term sustainability [23,69,77].

A favorable policy landscape further strengthens feasibility. Environmental health has moved to the center of European and global public health strategies. Initiatives such as the Horizon Europe Mission Cancer, the European Cancer Plan, and exposome-science consortia provide strategic alignment and funding pathways that directly support ambiomic approaches [78,79,80,81].

Nevertheless, challenges must be acknowledged. Training and clinical culture are central barriers; integrating environmental and social layers requires new competencies and professional profiles (environmental health nurses, exposome analysts, community navigators) [24,82,83,84]. Rapidly evolving treatment landscapes add further complexity: novel immunotherapy approaches—including monoclonal antibodies, chimeric antigen receptor (CAR) T cell therapy and immune checkpoint inhibitors—introduce distinct patterns of acute and late toxicities (e.g., B-cell aplasia, autoimmune complications) that demand flexible, adaptive follow-up programs and anticipatory interventions [85]. Interoperability also remains a key technical challenge as AHC requires the integration of environmental, clinical, genomic, geospatial, and behavioral data across heterogeneous electronic systems [86]. Implementing AHC will also require robust data governance and privacy safeguards, as well as explicit attention to the digital divide, so that data-intensive tools do not inadvertently widen existing inequities.

Financial sustainability is another consideration. Although the model builds on existing structures, long-term implementation requires stable investment in workforce, digital platforms, and community partnerships. Contextual variability adds further complexity: environmental exposures differ substantially across regions, meaning that scaling requires both fidelity to core principles and adaptation to local realities [78,81].

These opportunities—clinical maturity, policy alignment, scalability, and equity—contrast with real but surmountable challenges. Recognizing both dimensions ensures that the model can grow responsibly, effectively, and sustainably. Early international reception has also been encouraging: elements of the ambiomic survivorship model were first presented by J.A. Ortega-García at the 2nd Childhood Cancer Prevention Symposium held at Texas Children’s Hospital in Houston, Texas, in February 2025, where the approach was received with substantial interest as a scalable way of integrating environmental health into pediatric oncology care.

## 8. Future Directions: Advanced Exposomics and the Rise of Ambiomic Medicine

For decades, precision medicine in oncology has been driven primarily by genomics [87,88]. This revolution has sharpened diagnosis, refined risk groups, and guided targeted therapies. Yet genomic information alone cannot explain the full variability of treatment response, toxicity, relapse, or late effects—especially in children and adolescents, whose biology is continuously shaped by their surrounding environments. Health does not unfold in a vacuum: air and water quality, household and industrial chemicals, built environments, nutrition, stress, sleep, digital ecosystems, social networks, and climate-related factors interact dynamically with the genome across the life course [89,90].

Classical pediatric tumor markers (AFP, β-hCG, HVA/VMA, LDH, ALP) largely reflect late biological consequences and often remain within normal ranges during early stages of childhood cancers. Within an ambiomic framework, these biomarkers are interpreted as downstream effects of cumulative gene–environment perturbations rather than as early detection tools. The development of anticipatory biomarkers integrating exposomic, genomic, epigenomic, metabolic and microbiome-derived signals—supported by AI-based pattern recognition—represents a critical frontier for future survivorship care. Such multi-omic markers may identify pre-disease trajectories well before conventional tumor markers change, enabling earlier intervention and personalized risk navigation within survivorship programs.

Advanced exposomics addresses this gap by systematically characterizing the totality of environmental exposures—chemical, physical, biological, and social—and linking them to molecular and clinical trajectories [63,90]. By transforming static exposure categories into living exposure landscapes, exposomics enables clinicians to anticipate complications, tailor interventions, and incorporate environmental and social intelligence into routine care. This shift is especially urgent for cancer survivors, who live long lives shaped not only by their genetic legacy and treatment history, but also by the quality of the environments they inhabit [5,6,7,8,9,25].

A rapidly emerging field—mutational epidemiology—may further strengthen this transition [91,92]. Taken together, advanced exposomics and mutational epidemiology move the field beyond genomics-only precision—toward an ambiomic model where prevention is as precise as treatment [25]. By identifying exposure-specific mutational signatures and their epigenetic correlates, mutational epidemiology makes it possible to decode how specific agents contribute to tumorigenesis, toxicities, and long-term outcomes [91,92,93,94,95]. When combined with exposomic surveillance, this approach opens the door to an integrative, exposure-aware precision medicine that unites environmental epidemiology, molecular oncology, and prevention [18,25,60,91,92,93,94,95]. Partnerships with genetic counselors create an upstream, exposure-aware pathway into ambiomic care for families with hereditary risk [61,96,97].

**Figure 2:**
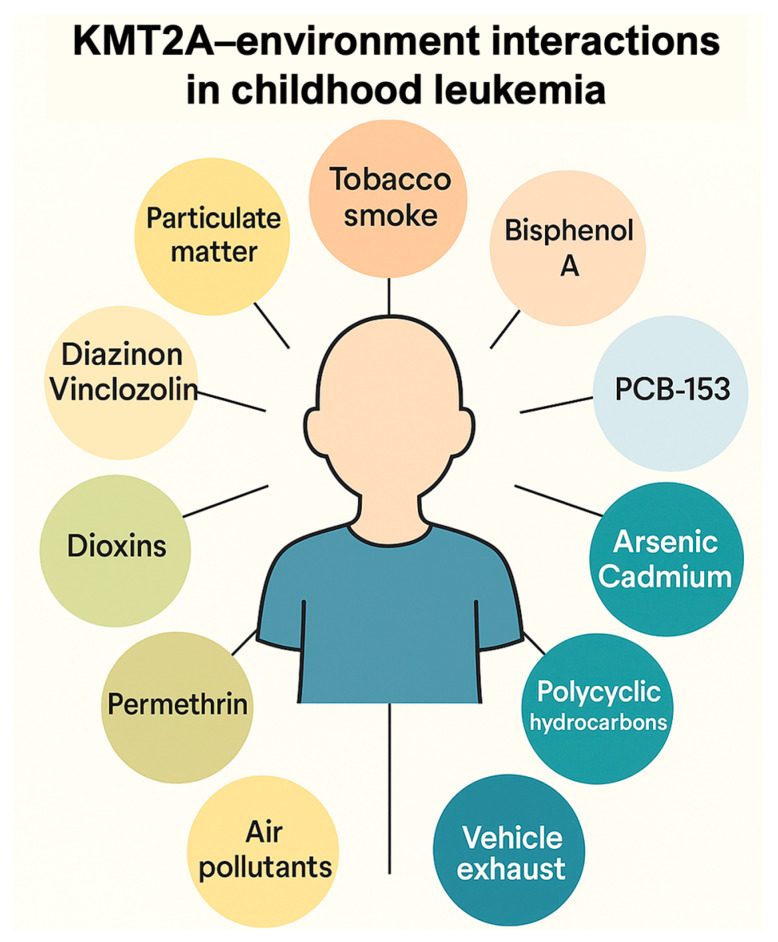
**Example of ambiomic mechanistic insight: KMT2A–environment interaction network derived from the Comparative Toxicogenomics Database (CTD).** The diagram shows selected environmental chemical interactors recurrently linked to KMT2A across CTD inference networks for “Leukemia”, “Precursor Cell Lymphoblastic Leukemia–Lymphoma”, “Cell Transformation, Neoplastic” and “Neoplasms”, grouped into major exposure domains (air pollutants and particulate matter, tobacco smoke and vehicle exhaust, metals and metalloids such as arsenic and cadmium, pesticides including diazinon and vinclozolin, and endocrine-disrupting/industrial chemicals such as bisphenol A and dioxins). These curated relationships illustrate how gene–environment networks can inform anticipatory, exposure-aware clinical decisions within ambiomic survivorship models. Infant KMT2A-rearranged leukemias are a paradigmatic model of gene–environment interaction: most arise prenatally, are driven by KMT2A fusion proteins that reprogram HOX-dependent transcription, and may be modulated by in utero and early-life environmental exposures. Source: Prepared by the authors.

To illustrate how ambiomic tools can translate mechanistic knowledge into anticipatory care, we derived a KMT2A–environment interaction network using the Comparative Toxicogenomics Database (CTD) (Figure 2). Starting from CTD inference networks that link KMT2A to “Leukemia”, “Precursor Cell Lymphoblastic Leukemia–Lymphoma”, “Cell Transformation, Neoplastic” and “Neoplasms”, we curated a set of recurrent environmental chemicals interacting with KMT2A. These include major air- and combustion-related exposures (air pollutants and particulate matter, tobacco smoke pollution, vehicle emissions), metals and metalloids (arsenic, cadmium), pesticides (e.g., diazinon, vinclozolin), and endocrine-disrupting or industrial chemicals (bisphenol A, dioxins, selected polychlorinated biphenyls). Together, they outline an ambiomic gene–environment network centered on KMT2A. Infant KMT2A-rearranged leukemias are a paradigmatic model of such interactions: most arise prenatally, are driven by KMT2A fusion proteins that reprogramme HOX-dependent transcription, and may be modulated by in utero and early-life environmental exposures. In clinical practice, similar networks can be used to prioritize exposure assessment, biomonitoring and preventive counseling for children treated for KMT2A-driven malignancies. This gene–environment interaction map, derived from the CTD, demonstrates how curated toxicogenomic data can reveal exposure-sensitive pathways relevant to risk stratification, toxicity anticipation, and personalized intervention planning [98]. Such mechanistic insights exemplify how ambiomic analytics can complement clinical judgment, especially when integrated into the AHC.

This integrative paradigm lays the foundation for Ambiomic Medicine: a next-generation model that unifies genomic, epigenomic, exposomic, behavioral, and geospatial data into a single interpretive system—the ambiome [18]. Like a conductor harmonizing multiple instruments, the ambiome connects genetic information with environmental signals to shape biological responses and clinical decisions in real time. Ambiomic tools are not merely descriptive; they are anticipatory [64,99]. They allow clinicians to act on modifiable exposures from day zero, reducing treatment-related mortality, preventing complications, and modulating chronicity. Critically, ambiomic survivorship is designed to integrate emotional and mental health burden—such as anxiety, depression and post-traumatic symptoms—alongside physical late effects, so that navigation and interventions address the full complexity of life after cancer.

This evolution also mirrors a broader scientific and societal transition. In the context of the Anthropocene—where climate change, pollution, biodiversity loss, and urban stressors increasingly shape pediatric health—exposomic intelligence and ambiomic medicine align with planetary health principles [100,101,102]. By 2030, it is conceivable that survivorship clinics will routinely integrate exposomic dashboards; that environmental risk stratification will be as common as genomic sequencing; and that prevention will be as personalized as treatment. Ambiomic medicine does not replace precision medicine—it completes it—embedding treatment and survivorship within a living ecological context. At the European level, this ambiomic survivorship vision has inspired the AIREACH proposal (Air Quality and Integrated Risk Evaluation for Childhood, Adolescent and Young Adult Cancers), a multicentre consortium coordinated by Hospital Sant Joan de Déu (Barcelona) and submitted to the EU Mission Cancer call HORIZON-MISS-2025-02-CANCER-02 [80].

Looking forward, the impact of ambiomic survivorship models can be measured through a structured set of short-, medium-, and long-term indicators, including reductions in treatment-related mortality and acute complications; faster functional recovery and quality of life; exposure reduction; improved equity; and demonstrable cost-effectiveness [70,79]. Table 4 summarizes expected outcomes across 12, 24, and 60 months, providing a roadmap for real-world implementation and evaluation.

Co-design with survivors and families, including systematic use of PROs and PREMs, will be essential to ensure that ambiomic survivorship models truly reflect lived experience and priorities.

Because most of the data and examples presented here derive from a single regional program, multicenter validation and adaptation in diverse health systems, including low- and middle-income settings, will be critical to confirm feasibility, scalability and impact.

## 9. Conclusions

Pediatric and AYA cancer survivorship has reached a turning point. We can no longer rely solely on post-treatment surveillance designed for a different era. The scientific and social context has changed: survival is longer, vulnerability is dynamic, and environmental determinants play a decisive role in shaping outcomes [72,81].

The PEHis–AHC model offers a practical and visionary response. Rooted in real-world clinical practice, it shows that integrating environmental and community intelligence into survivorship care improves outcomes, strengthens systems, and empowers families [5,6,7,23,57,58,68]. It does not seek to replace existing guidelines but to expand and enrich them, turning static follow-up into a living, anticipatory process that begins at diagnosis.

The next frontier is clear. Advanced exposomics and ambiomic medicine will allow us to understand and act on the full landscape of risk and resilience—merging genome and epigenome, exposome, and society [61,63,90]. This is the future of precision: not just targeted treatment, but targeted prevention, equity, and planetary health [18,91]. Ambiomic medicine expands precision medicine by unifying genomic signals with environmental and social determinants, converting static follow-up into real-time, exposure-aware navigation from diagnosis [25].

The opportunity is within reach. What began in one region can become a blueprint for Europe and beyond. To seize it, we must act together—clinicians, scientists, policymakers, and communities—to ensure that the next generation of cancer survivors not only lives longer, but lives better.

## Figures and Tables

**Table 1:** From the current model to an ambiomic, proactive approach from day zero.

Dimension	Current Model (Late, Reactive)	Proposed Ambiomic Approach (Early, Proactive)	Expected Impact
**Timing**	Follow-up activated after treatment	Begins at diagnosis (Day 0), accompanies the full continuum	↓ TRM; ↓ preventable events during therapy
**Risk stratification**	Static (dose and organ based)	Dynamic (genomic–environmental–social layers with recalibration)	Better discrimination and timely decisions
**Intervention logic**	Periodic screenings and referrals	Clinical–environmental package + proactive alerts	↓ severe infections; ↓ ICU admissions; ↓ readmissions
**Decision timing**	Calendar-based visits	Real-time, context-driven decisions	Faster response to risk peaks
**Care integration**	Fragmented across specialties	Hospital–primary care–public health–schools	Continuity and safer transitions
**Data & measurement**	Biomedical focus	Clinical–environmental–social layers + PROMs/PREMs + KPIs	Transparency and continuous improvement
**Diagnostic radiation**	Cumulative use, rarely audited	Low-exposure pathways and monitoring	↓ annual effective dose
**Data architecture**	Heterogeneous systems; poor interoperability	Integrated clinical–environmental–geospatial data flow	Better desision support and reproductibility
**Pediatric–Adult** **Transition**	Irregular handover	Guided pathway with case manager	↓ loss to follow-up
**Equity**	Generic mentions	Stratified outcomes + social prescribing	↓ outcome gaps by vulnerability
**Population Goal**	Maintain surveillance	≥90% 5-year survival, TRM → toward lowest achievable (“functional zero”)	improved long-term outcomes
**Sustainability &** **social value**	Fragmented delivery	Integrated pathways with community and public health	Greater social return and health efficiency

Note: TRM = treatment-related mortality; PROMs/PREMs = patient-reported outcome/experience measures; KPIs = key performance indicators. Downward arrows (↓) indicate a reduction or decrease in the corresponding outcome.

**Table 2:** Added value of PEHis and AHC compared with conventional survivorship guidelines.

Dimension	International Guidelines (COG/IGHG/PanCare/NCCN)	Pediatric Environmental History (PEHis)	Ambiomic Health Compass (AHC)
Timing of survivorship care	Activated post-treatment; calendar-based surveillance	Initiated at diagnosis; integrates environmental and social risk	Day-0 activation; dynamic recalibration and real-time alerts
Risk framework	Static, chemotherapy/radiation dose/organ-based	Adds contextual environmental and social determinants	Dynamic ambiomic risk (genomic–exposomic–social) with continuous updating
Environmental layer	Limited lifestyle recommendations	Systematic environmental history (Green Page/Green Passport)	Exposomic intelligence: air, water, pollutants, housing, radon, school environments
Social determinants	Mentioned but not structured	Structured assessment of family and community factors	Predictive social vulnerability scoring + social prescribing pathways
Care integration	Specialty-centered	Bridges oncology–primary care–public health–schools	Integrated navigation system with shared digital plan
Monitoring tools	Organ-specific tests	Environmental and behavioral monitoring	Contextual alerts + geospatial data + biomarkers
Predisposition clinics (hereditary cancer)	Mentioned; however, handled by separate pathways/speciality	Adds structured environmental and social profile for families with germline risk; household/school actions	Toxicogenomic mapping + targeted biomonitoring; radiation-sparing surveillance integration; cascade testing workflow; shared plan with genetics
Biomonitoring	Not included	Not systematic	Targeted biomonitoring (metals, pesticides, tobacco metabolites) linked to vulnerability
Toxicogenomics/gene–environment mapping	Not included	Not included	Incorporates toxicogenomic interaction maps (e.g., CTD), enabling personalized anticipatory interventions
Transition (child → AYA → adult)	Variable implementation	Structured environmental handover	Guided longitudinal transition with data continuity and explicit roles
Equity	Limited	Improves access through community involvement	Equity as explicit metric: outcomes stratified by vulnerability, rurality, migration

Note: PEHis = Pediatric Environmental History; AHC = Ambiomic Health Compass; GLS = global longitudinal strain; SPM = second primary malignancies; DXA = dual-energy X-ray absorptiometry. CTD = Comparative Toxicogenomics Database; COG/IGHG/PanCare/NCCN, PEHis, CTD, AYA.

**Table 3:** Core clinical and system tools: what they are, when they are used, and what they deliver.

Tool	Description	Timing of Use	Clinical Output	Associated Metrics
**PEHis (Green Page/Passport)**	Structured environmental and social history linked to a living care plan	D0–D + 30 and follow-up	Personalized plan and family education	PEHis coverage (%); active plan at D + 30 (%)
**Clinical–Environmental Package (MRT-0)**	Prophylaxis, clean air, radon mitigation, sleep, activity, nutrition, vaccination, psychosocial support	During treatment	↓ infections and toxicity	Severe infections/100 cycles; ICU admissions/100
**Navigation & Social Prescription**	Case navigation linking clinic with school/municipality	Continuous and at transition	Barrier resolution and adherence	Visit/medication adherence; PREMs
**Home/School Environmental Measurement**	Simple IAQ, ventilation, filtration, radon screening, risk-based recommendations	D0 and risk reassessment	Exposure reduction	Smoke-free homes (%); radon mitigated (%)
**Essential Biomarkers**	Cotinine, selected metabolites, metabolic profile, vitamin D	Baseline and clinical decision points	Plan confirmation and adjustment	% biomarkers with actionable intervention
**Ambiomic Analytics (AHC)**	Dynamic risk, explainable alerts and recommendations	From D + 14 onward	Real-time actionable decisions	AUC/net benefit; alerts resolved < 14 d
**Clinical/Family Dashboard and KPIs**	Risk, actions and outcomes visualization	Continuous (quarterly audit)	Traceability and improvement	KPI adherence (%); data latency (days)
**Longitudinal Registry 0–39**	Integration with primary care and public health	Continuous	Seamless continuity of care	Loss to follow-up (%); data completeness (%)
**Ecological Informed Consent**	Environmental dimension in shared decisions	D0 and key reviews	Patient engagement and understanding	% consents with environmental component
**Economic Evaluation**	Cost-effectiveness and ROI	Annual	Sustainability and scaling	ICER (€/QALY); ROI at 5 years

Note: IAQ = indoor air quality; AHC = Ambiomic Health Compass; KPI = key performance indicator; ROI = return on investment; ICER = incremental cost-effectiveness ratio; PEHis = Pediatric Environmental History; TRM = Treatment-Related Mortality; ICU = Intensive Care Unit; AUC = Area Under the Curve; PREMs = Patient-Reported Experience Measures. The arrow (↓) indicates a reduction in clinical toxicity and infection frequency associated with the intervention.

**Table 4:** Metrics and expected outcomes across time horizons.

Horizon	Clinical	Experience & Function	Exposure & Resource Use	Equity & Risk Management	Value/Cost-Effectiveness
**12 months**	TRM (% and per 1000 pt-years); severe infections/100 cycles; ICU admissions/100; avoidable readmissions	PROMs (fatigue, mood); time to return to school/daycare; family satisfaction	Diagnostic radiation dose (mSv); avoidable hospital days; vaccination adherence	Outcomes stratified by SES/rurality/migration; transition losses	Intervention cost vs. savings from preventable events
**24 months**	Subclinical cardiotoxicity (GLS/E/e′); unplanned visits and readmissions; metabolic control	Stable HRQoL; sustained school/work attendance; neurocognitive improvement	Cumulative radiation reduction; rational use of testing	Improved adherence and reduced outcome gaps; social prescription coverage	Preliminary ICER (€/QALY); positive ROI trend
**60 months**	5- and 10-year survival; SPM incidence (SIR); chronic comorbidity burden	Full educational/work participation; sustained mental health	Optimized health resource use; seamless transition	Convergence of outcomes across vulnerable and non-vulnerable groups	Definitive ICER; ROI ≥ 1; budgetary sustainability

Note: TRM = treatment-related mortality; PROMs = patient-reported outcomes; SES = socioeconomic status; GLS = global longitudinal strain; HRQoL = health-related quality of life; SPM = second primary malignancies; ROI = return on investment; ICER = incremental cost-effectiveness ratio; mSv—millisievert; QALY = quality-adjusted life year; SIR = standardized incidence ratio.

## Data Availability

No new data were created or analyzed in this study. Data sharing is not applicable to this article.

## Abbreviations

The following abbreviations are used in this manuscript:

CCS	Childhood cancer survivors
AYA	Adolescent and Young Adult
PEHSU	Pediatric Environmental Health Specialty Unit
PEHis	Pediatric environmental history
AHC	Ambiomic Health Compass
TRM	Treatment-related mortality
QoL	Quality of life
IAQ	Indoor air quality
COG	Children’s Oncology Group
IGHG	International Late Effects of Childhood Cancer Guideline Harmonization Group
PanCare	Pan-European Network for Care of Survivors after Childhood and Adolescent Cancer
NCCN	National Comprehensive Cancer Network
PAHs	Polycyclic Aromatic Hydrocarbons
WHO	World Health Organization
CTD	Comparative Toxicogenomics Database
PROs	Patient-reported outcomes
PREMs	Patient-reported experience measures
